# Early detection of a circulating pre-vaccine-derived poliovirus type 1 (pre-VDPV1) variant linked to an acute flaccid polio case prior to VDPV1 emergence, Israel, 2024 to 2025

**DOI:** 10.2807/1560-7917.ES.2025.30.34.2500605

**Published:** 2025-08-28

**Authors:** Neta S Zuckerman, Yaniv Lustig, Danit Sofer, Lester M Shulman, Leah Weiss, Rinat Vasserman, Reut Gabai, Keren Friedman, Hagar Eliyahu, Noa Bar-Liss, Tatyana Kushnir, Ira Aguvaev, Tal Butensky, Daniel Avhar, Alex Aydenzon, Oran Erster, Itay Bar-Or, Merav Weil

**Affiliations:** 1 Central Virology Laboratory, Public Health Services, Ministry of Health, Chaim Sheba Medical Center, Ramat Gan, Israel; 2 School of Public Health, Gray Faculty of Medicine and Health Sciences, Tel-Aviv University, Tel-Aviv, Israel

**Keywords:** Vaccine Derived Polio Virus (VDPV), Vaccine-associated paralytic poliomyelitis (VAPP), Polio type 1 (PV1), Environmental surveillance, Whole Genome Sequencing (WGS), Acute Flaccid Paralysis (AFP)

## Abstract

We report the emergence and evolution of a circulating vaccine-derived poliovirus type 1 (cVDPV1) outbreak in Israel, linked to a vaccine-associated paralytic poliomyelitis case. Whole genome sequencing revealed a strong genetic link between the Sabin-like poliovirus type 1 variant from the case and pre-VDPV1 and VDPV1 isolated from environmental samples collected in October 2024–April 2025, mostly in Jerusalem. Early detection was made possible by Israel’s robust environmental surveillance and advanced sequencing technologies, enabling a rapid public health response.

Vaccine-derived polioviruses (VDPV) can cause paralysis and outbreaks, posing a major challenge to public health and eradication efforts [1]. Recently, circulating VDPV (cVDPV) outbreaks have emerged worldwide, including in high-income countries [2-5]. Herein, we describe how environmental surveillance and advanced sequencing technologies in Israel enabled early detection of genetically related Sabin-like poliovirus type 1 (SL1) variants from a common source, linked to an acute flaccid paralysis (AFP) case, which subsequently evolved into circulating VDPV type 1 (cVDPV1).

## Detection of Sabin-like poliovirus type 1 in an acute flaccid paralysis case

In late December 2024, an unvaccinated adolescent from an ultra-orthodox family in Jerusalem was hospitalised with AFP. Three of the patient’s samples (two stool samples, collected one day apart, and a throat swab) tested positive for SL1 using the polio isolation method [6,7]. Sanger sequencing of the VP1 region revealed 4 to 7 nucleotide substitutions relative to the Sabin1 vaccine strain, not meeting the > 10 mutations criteria to be classified as a VDPV1 [8]. The nucleotide substitutions identified in the VP1 region of poliovirus isolates sequenced via Sanger sequencing are provided in Supplementary Table S1. Follow-up stool and throat swab samples from the patient collected 2 weeks later and stool samples from seven close contacts tested negative for poliovirus.

## Investigation of the source of Sabin-like poliovirus type 1 detected in the case

Rare paralysis cases from the oral polio vaccine (OPV) are termed vaccine-associated paralytic poliomyelitis (VAPP) [9]. Since the patient was unvaccinated, exposure was presumed to be through contact with a recently OPV-vaccinated individual. However, no such source was identified despite thorough epidemiological investigation. Thus, potential exposure through an ongoing asymptomatic community circulation of an SL1 variant was investigated using Israel’s national polio environmental surveillance system.

As part of the investigation, VP1 region Sanger sequences from SL1 and VDPV1 isolates from 197 wastewater samples collected at 27 different sampling sites between October 2024 and April 2025 were compared with the Sabin 1 vaccine strain (AY184219.1) and the VAPP case isolates. Three substitutions found in the VAPP case (T306C, A316G, and C753T; Supplementary Table S1) were also found in 67 of the environmental isolates, suggesting the emergence of a specific SL1 variant linked to the VAPP case which is likely circulating.

The Table summarises SL1 detections at sampling sites over time. The SL1 variant was initially detected in the Jerusalem region and remains prevalent there, including in Beit Shemesh (n = 9), Kidron (south-east Jerusalem, n = 7), Og (north-east Jerusalem, n = 5) and Sorek (west Jerusalem, n = 3). Three VDPV1 isolates, each with 10–12 substitutions, were later detected in the Jerusalem area, in Kidron (Feb), Og (Apr) and Beit Shemesh (Apr). From January 2025, the SL1 variant spread to central Israel, and from March to southern and northern Israel, though at lower frequencies to date.

**Table:** Sabin-like poliovirus type 1 variant and vaccine-derived poliovirus type 1 detections in wastewater surveillance, Israel, October 2024–March 2025 (n = 27 sampling sites)

Sampling site	Estimation of population size	Epidemiological weeks																												
		2024												2025																
		Oct				Nov				Dec				Jan					Feb				Mar				Apr			
		41	42	43	44	45	46	47	48	49	50	51	52	1	2	3	4	5	6	7	8	9	10	11	12	13	14	15	16	17
**North district**																														
El-Hamra	22,120											neg				neg			neg				neg				neg			
Zafed	39,000				neg	neg					neg		neg		neg				neg				neg		**V**					neg
Haifa	741,050				neg				Neg	neg						neg				neg					neg	neg		neg		neg
Acre^a^	172,800														neg					neg				neg					neg	
Tiberias^a^	47,000															neg				neg			neg			neg		neg		
Maale Eron^a^	155,900																			neg										neg
**Central district**																														
Netania	303,000				neg							neg			neg				neg				**V**				neg		neg	
Shafdan	2,401,000		neg			neg					neg				neg						**V**		**V**		neg		**V**			
Ayalon	367,000		neg								neg				neg						**V**		neg			neg	**V**			
Drom HaSharon^a^	82,450	neg				neg										neg				neg				neg				neg		
Kfar Saba^a^	168,000	neg				neg						neg				neg			neg				**V**				**V**		**V**	
Elad^a^	51,400															neg				neg			neg		neg		neg		**V**	
Bnei Brak^a^	50,300													**V**		neg				neg				neg			neg		**V**	
Rishon LeZion^a^	269,800																	neg		neg		neg	neg		neg		neg			neg
Ramle Lod^a^	187,800															neg				neg				neg				neg		
Modiin-Illit^a^	83,700													neg		**V**				neg			neg			neg		neg		
**Jerusalem district**																														
Kidron	301,600							**V**						**V**		**V**		**V**		**V**		**VDPV1**		neg		neg		**V**		**V**
Jerusalem Og	213,000	neg						neg		neg						**V**		**V**		**V**				**V**		**V**		neg		**VDPV1**
Jerusalem Sorek B	669,094	neg							Neg			**V**		neg		neg		**V**		neg			neg		neg	**V**		neg		neg
Beit Shemesh	193,900		**V** ^b^					neg		**V**		**V**		**V**		**V**			**V**		**V**		**V**		**V**		**V**		**VDPV1**	
Jerusalem Refaim B^a^	464,000											neg				neg				neg			neg		neg			neg		neg
**South district**																														
Beer-Sheva	290,700		neg					neg		neg						neg			neg				neg				neg			
Rahat	83,700		neg					neg		neg				neg		neg			neg				neg							neg
Arara	21,000				neg			neg		neg						neg			neg					neg				neg		
Ashkelon	169,500		neg				neg			neg						neg			neg				neg						neg	
Ashdod	231,200	neg						neg		neg						neg			neg				**V**		**V**		neg			
Dimona^a^	42,000				neg			neg		neg						neg			neg		neg		neg		neg		neg		neg	

neg: negative; SL1: Sabin-like poliovirus type 1; V: SL1 variant; VDPV: vaccine-derived poliovirus.

^a^ These sites were gradually incorporated into routine surveillance and may not have been sampled earlier in the study period. The first result provided corresponds to the initial sampling event where a result (negative or positive) was recorded at the newly sampled location.

^b^ Indicates a pre-SL1 variant identified via ONT sequencing (see section ‘Whole genome sequencing broadens genetic links among SL1 isolates’).

Detections in sampling sites are marked with ‘neg’ (sampled but no SL1 variant detected), bold ‘V’ (SL1 variant detected) or ‘VDPV1’ (SL1 variant detected and identified as VDPV1, with > 10 VP1 mutations per GPEI guidelines [8]). Sites with SL1 or VDPV1 variant detections are indicated with blue shading. Estimated population size is listed for each site.

According to Global Polio Eradication Initiative (GPEI) guidelines [8], two genetically linked VDPV1 isolates from non-overlapping regions indicate a cVDPV outbreak. In the current event, three VDPV1 isolates were identified from two distinct regions (Beit Shemesh and Kidron/Og), with a genomic link between them and to environmental pre-VDPV1 and the VAPP case isolates. However, VP1 sequencing revealed only a weak three-mutation signature across the isolates, with greater divergence than similarity within the VP1 region. Thus, VP1 data alone were insufficient to classify the event as cVDPV1.

## Whole genome sequencing broadens genetic links among Sabin-like poliovirus type 1 isolates

**Figure 1:**
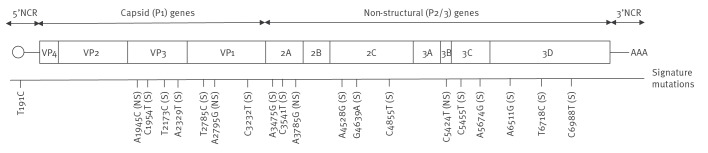
Genomic structure and mutations of the Sabin-like poliovirus type 1 variant, Israel, October 2024–April 2025

NCR: non-coding region; SL1: Sabin-like poliovirus type 1; VDPV: vaccine-derived poliovirus.

To better establish the genetic signature linking the SL1 variant isolates, whole genome sequencing (WGS) was applied using the SMARTer Stranded RNA-Seq kit (Takara Bio) and Illumina MiSeq (Illumina). Reads were aligned to Sabin 1 (AY184219.1) and consensus sequences were generated (60% threshold). Isolates with > 90% coverage were included in the analysis. Whole genome sequencing of environmental (n = 58) and clinical SL1 variant-positive isolates (n = 5) and additional non-variant SL1 isolates (n = 5) collected from various regions during the outbreak period, revealed a distinct 20-mutation signature across the genome. These included mutations in the VP1 (n = 3) and VP3 (n = 4) capsid regions and in non-capsid coding and non-coding regions (n = 13). One of the signature mutations in VP1, A2795G, occurred at an attenuation site and represents a reversion to wild-type PV1. One additional mutation, G480A in the 5'NCR, strongly associated with loss of attenuation [10], was excluded from the outbreak signature as it appears in both the SL1 variant and non-variant isolates in this study (Figure 1 and Supplementary Table S2, which details nucleotide and amino acid mutations identified in whole genome poliovirus sequences ).

Signature mutations shared across all SL1 variant and VDPV1 isolates are shown according to the genomic regions in which they occur. Each mutation is annotated as nonsynonymous (NS) or synonymous (S).

**Figure 2:**
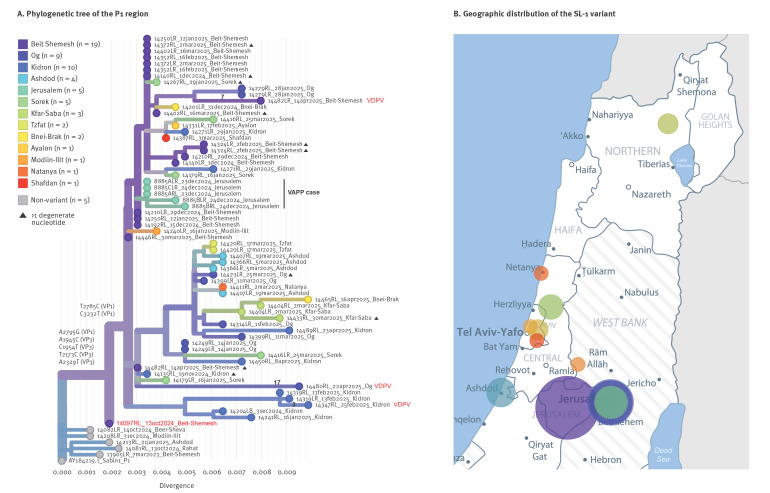
Phylogenetic analysis of the P1 capsid region of the Sabin-like poliovirus type 1 variant, Israel, October 2024–April 2025 (n = 68 sequenced isolates)

SL1: Sabin-like poliovirus type 1; VAPP: vaccine-associated paralytic poliomyelitis; VDPV: vaccine-derived poliovirus.

To explore viral evolution and community links, we performed phylogenetic analysis using the Nextstrain pipeline [11] of the P1 region of all 68 sequenced isolates (Figure 2). The analysis showed a genetically diverse outbreak, with SL1 variant evolving into multiple distinct lineages across Israel. Most lineages spanned multiple locations, except one unique to the Kidron site. Interestingly, each of the three VDPV1 detections was associated with a different lineage. The Kidron isolate (specimen ID 14347RL) clustered with viruses detected only in Kidron. The Beit Shemesh isolate (specimen ID 14482LR) grouped with viruses from Og, Beit Shemesh and Bnei-Brak. In contrast, the Og isolate (specimen ID 14480RL) belonged to a defined cluster but carried an unusually high number of unique mutations (n = 17), including seven nonsynonymous mutations within the P1 region (Supplementary Table S2). The VAPP case pre-VDPV1 isolates were related to the Beit Shemesh VDPV1 cluster.

A. Phylogenetic tree of the P1 region (VP1–VP4 genes; 2,643 nt) showing genomic differences among the samples (n = 68). The 'n' indicated next to each sampling site refers to the number of sequenced isolates obtained from each site. Tree nodes are colour-coded by the location of detection. Labels next to each node include the sample name, detection date and location. The VAPP case isolates are indicated (n = 5). Isolates identified as VDPV1 are marked in red, with the number of mutations relative to their nearest split indicated. The signature mutations shared by all outbreak-related isolates are marked on the branches defining the outbreak cluster (positions are relative to Sabin1 whole genome, AY184219.1). The earliest detected outbreak-related isolate (specimen ID: 14097RL) is marked in red. Sequences containing more than one ambiguous nucleotide and lacking sufficient resolution for sub-lineage classification beyond their current placement are marked with a triangle (►).

B. The map shows the geographic distribution of detections across Israel. Map circles represent regions of SL1 variant detection, with colour indicating geographic location and size proportional to the number of SL1-V sequences from each location. The environmental surveillance data presented here refer to the state of Israel only and do not include the West Bank or the Gaza Strip.

As a proof of concept, we re-sequenced the earliest SL1 variant isolate detected in Beit Shemesh, the initial outbreak detection area (specimen ID 14097RL, 13 Oct 2024), using a MinION device (Oxford Nanopore Technologies (ONT) [12]), enabling complete genome sequences. Sanger sequencing previously indicated a mixed base (R = A/G) at one of the SL1 variant signature mutations VP1:A316G, suggesting a mixture of SL1 non-variant and pre-SL1 variant genomes, possibly resulting in a consensus sequence that reflected the non-variant strain and did not cluster with the outbreak. ONT sequencing, combined with downstream bioinformatic clustering analysis, allowed the resolution of complete genomes from the heterogeneous sample. Genomes with 15/20 SL1 variant signature mutations were identified, suggesting this isolate might represent an early SL1 variant form, before acquiring five additional mutations, including two in VP1 (Figure 2).

## Discussion

This study was initiated following the detection of SL1 in Israel in 2024 in an AFP isolation from an unknown source, prompting further investigation of environmental samples for potential circulation. Findings indicate the emergence, evolution and widespread circulation of SL1 variant in Israel, primarily concentrated in the Jerusalem region [5]. In the past 4 years, Israel experienced two additional VDPV outbreaks: type 3, which remained localised, and type 2, which spread internationally. Similar to the current outbreak, both of these outbreaks originated in Jerusalem and expanded to other cities with large ultra-orthodox populations; each included one AFP case [4,5].

Globally, VDPV type 1 is the second most commonly reported strain, though far less than type 2. Between January 2023 and June 2024, cVDPV1 circulation was detected in three African countries (Democratic Republic of the Congo, Madagascar and Mozambique) [13], resulting in a high number of confirmed AFP cases (n = 140). The VDPV1 detected in Israel is unrelated to the African strains and appears to be locally derived, having evolved within the population. It was detected at an early stage, has so far been identified exclusively in Israel, and likely originated from routine administration of the Sabin 1 OPV. 

These outbreaks highlight ongoing challenges to polio eradication efforts, particularly where immunisation coverage is sub-optimal. Whole genome sequencing was instrumental in enhancing the resolution of the outbreak’s genetic profile, uncovering a strong genetic linkage of 20 shared mutations across all clinical and environmental SL1 variant and VDPV1 isolates which was not possible using the VP1 region alone. Most importantly, this enhanced linkage among the three distinct VDPV1 detections, of which two were from non-overlapping regions, providing the evidence needed to characterise this event as a cVDPV1 outbreak. Similarly, WGS enabled early VDPV2 outbreak detection in Israel’s previous outbreak, despite just two shared VP1 mutations [14]. These findings underscore the importance of incorporating WGS into routine poliovirus surveillance to enhance genetic linkage and enable early detection.

Interestingly, mutation analysis of the Og VDPV1 isolate (14480RL) may suggest prolonged excretion by an immunodeficient individual. It exhibited a high number of unique mutations (n = 17) including seven P1-region nonsynonymous mutations mostly in VP1, consistent with individual viral evolution rather than person to person transmission [15]. 

Long-read ONT sequencing enabled the identification of a pre-SL1 variant sequence from a mixed early isolate with 15/20 SL1 variant signature mutations, providing the earliest evidence of an SL1 variant isolate during the study period. To our knowledge, this is the first use of advanced whole genome sequencing and analysis to resolve co-circulating genomes from homotypic mixtures in environmental samples, enabling high-resolution outbreak insights. 

## Conclusion

Integrated genomic analysis together with demographic and epidemiological data from Israel’s extensive environmental surveillance enabled early detection of genetically related SL1 variants, prior to their official classification as cVDPV1 under GPEI guidelines. Such timely identification is especially important in areas with unvaccinated populations and demographic conditions that facilitate virus transmission, allowing rapid, targeted public health interventions that helped prevent further cases. However, global insight into this outbreak remains limited, as many countries lack surveillance and whole genome sequencing at the scale achieved in Israel. Consequently, further international spread of the virus may remain undetected in early circulation stages.

## Data Availability

Sequences have been deposited in GenBank under accession numbers PV867330–PV867397.

## Supplementary Data

70000 Supplementary Table 1

## Supplementary Data

394000 Supplementary Table 2

## References

[1] Burki T . Vaccine-derived poliovirus cases exceed wild types. Lancet Infect Dis. 2019;19(2):140. 10.1016/S1473-3099(19)30012-X 31876508

[2] World Health Organization (WHO). Detection of circulating vaccine derived polio virus 2 (cVDPV2) in environmental samples– the United Kingdom of Great Britain and Northern Ireland and the United States of America. Geneva: WHO; 2022. Available from: https://www.who.int/emergencies/disease-outbreak-news/item/2022-DON408

[3] Böttcher S Kreibich J Wilton T Saliba V Blomqvist S Al-Hello H Detection of circulating vaccine-derived poliovirus type 2 (cVDPV2) in wastewater samples: a wake-up call, Finland, Germany, Poland, Spain, the United Kingdom, 2024. Euro Surveill. 2025;30(3):2500037. 10.2807/1560-7917.ES.2025.30.3.2500037 39850005 PMC11914958

[4] Zuckerman NS Bucris E Morad-Eliyahu H Weiss L Vasserman R Fratty IS Environmental surveillance of a circulating vaccine-derived poliovirus type 2 outbreak in Israel between 2022 and 2023: a genomic epidemiology study. Lancet Microbe. 2024;5(10):100893. 10.1016/S2666-5247(24)00116-2 39284332

[5] Weil M Sofer D Shulman LM Weiss L Levi N Aguvaev I Environmental surveillance detected type 3 vaccine-derived polioviruses in increasing frequency at multiple sites prior to detection of a poliomyelitis case. Sci Total Environ. 2023;871:161985. 10.1016/j.scitotenv.2023.161985 36739034

[6] World Health Organization (WHO). Polio Laboratory Manual. Geneva: WHO; 2004. Available from: https://iris.who.int/bitstream/handle/10665/68762/WHO_IVB_04.10.pdf

[7] World Health Organization (WHO). S1. Supplement to the WHO Polio Laboratory Manual. An alternative test algorithm for poliovirus isolation and characterization. Geneva: WHO; 2020. Available from: https://polioeradication.org/wp-content/uploads/2017/05/NewAlgorithmForPoliovirusIsolationSupplement1.pdf

[8] World Health Organization (WHO). Classification and reporting of vaccine-derived polioviruses (VDPV). Geneva: WHO; 2016. Available from: https://polioeradication.org/wp-content/uploads/2016/09/Reporting-and-Classification-of-VDPVs_Aug2016_EN.pdf

[9] Platt LR Estívariz CF Sutter RW . Vaccine-associated paralytic poliomyelitis: a review of the epidemiology and estimation of the global burden. J Infect Dis. 2014;210(Suppl 1) Suppl 1;S380-9. 10.1093/infdis/jiu184 25316859 PMC10424844

[10] Laassri M Dragunsky E Enterline J Eremeeva T Ivanova O Lottenbach K Genomic analysis of vaccine-derived poliovirus strains in stool specimens by combination of full-length PCR and oligonucleotide microarray hybridization. J Clin Microbiol. 2005;43(6):2886-94. 10.1128/JCM.43.6.2886-2894.2005 15956413 PMC1151934

[11] Hadfield J Megill C Bell SM Huddleston J Potter B Callender C Nextstrain: real-time tracking of pathogen evolution. Bioinformatics. 2018;34(23):4121-3. 10.1093/bioinformatics/bty407 29790939 PMC6247931

[12] Jain M Olsen HE Paten B Akeson M . The Oxford Nanopore MinION: delivery of nanopore sequencing to the genomics community. Genome Biol. 2016;17(1):239. 10.1186/s13059-016-1103-0 27887629 PMC5124260

[13] Namageyo-Funa A Greene SA Henderson E Traoré MA Shaukat S Bigouette JP Update on Vaccine-Derived Poliovirus Outbreaks - Worldwide, January 2023-June 2024. MMWR Morb Mortal Wkly Rep. 2024;73(41):909-16. 10.15585/mmwr.mm7341a1 39418214 PMC11486351

[14] Zuckerman NS Bar-Or I Sofer D Bucris E Morad H Shulman LM Emergence of genetically linked vaccine-originated poliovirus type 2 in the absence of oral polio vaccine, Jerusalem, April to July 2022. Euro Surveill. 2022;27(37):2200694. 10.2807/1560-7917.ES.2022.27.37.2200694 36111556 PMC9479469

[15] Burns CC Diop OM Sutter RW Kew OM . Vaccine-derived polioviruses. J Infect Dis. 2014;210(Suppl 1):S283-93. 10.1093/infdis/jiu295 25316847

